# Beyond the Skin: An Integrated Biopsychosocial Framework for Childhood Atopic Dermatitis

**DOI:** 10.3390/children13101359

**Published:** 2026-10-07

**Authors:** Hannia Bertha Matt Hernandez, Maria Isabel Rojo Gutierrez

**Affiliations:** 1 Allergy and Clinical Immunology, Hospital Christus Muguerza Reynosa, Reynosa 88610, Mexico; 2 Allergy and Clinical Immunology, Hospital Juarez de Mexico, Mexico City 07760, Mexico; mi_rojo@yahoo.com.mx

**Keywords:** atopic dermatitis, quality of life, psychosocial impact, sleep disturbance, caregiver burden

## Abstract

**Highlights:**

**What are the main findings?**

- Pediatric atopic dermatitis is associated with multidimensional psychosocial burden involving quality of life, sleep, emotional well-being, social functioning, and caregiver burden.
- Psychosocial impairment may be disproportionate to physician-assessed skin severity.

**What are the implications of the main findings?**

- Integrating biopsychosocial and burden-of-disease models provides a more comprehensive framework for assessing pediatric atopic dermatitis.
- A structured psychosocial assessment may facilitate patient- and family-centered care in routine clinical practice

**Abstract:**

Atopic dermatitis (AD) is a common chronic inflammatory skin disease in childhood characterized by pruritus, skin barrier dysfunction, and a relapsing–remitting course. Beyond its cutaneous manifestations, AD may substantially affect the psychosocial well-being of children and their families. Objective: To synthesize current evidence on the psychosocial burden of pediatric AD and integrate biopsychosocial and burden-of-disease frameworks into a clinically oriented approach, with particular relevance to pediatric allergy practice. Methods: A narrative review of literature published from January 2013 through May 2026 was conducted using PubMed/MEDLINE, Scopus, and Web of Science. Evidence addressing health-related quality of life, sleep disturbance, emotional and behavioral outcomes, social functioning, treatment-related burden, and caregiver and family burden was synthesized narratively, prioritizing pediatric populations. Results: Pediatric AD is associated with multidimensional burden involving health-related quality of life, sleep, emotional well-being, social functioning, and family life. Physician-assessed disease severity and patient- or family-perceived burden show imperfect correspondence, supporting the complementary use of patient- and caregiver-reported outcomes. Nocturnal pruritus and sleep disruption may contribute to bidirectional interactions among skin symptoms, emotional distress, and family burden. Visible lesions may contribute to stigma and social difficulties, while caregivers may experience sleep loss, stress, treatment burden, and concerns regarding topical corticosteroid use. Current guidance increasingly supports assessment of patient- and family-reported burden alongside objective disease severity. Conclusions: Integrating biopsychosocial and burden-of-disease perspectives may support a more comprehensive approach to pediatric AD. Routine care should include discussion of sleep, emotional well-being, social functioning, and family burden, with further assessment or multidisciplinary support considered when clinically relevant concerns are identified.

## 1. Introduction

### From a Skin Disease to a Biopsychosocial Phenomenon

Atopic dermatitis (AD) is one of the most common chronic inflammatory diseases in children [1] and is a common cause of medical consultation, healthcare resource utilization, and family burden [2]. It is characterized by severe pruritus, xerosis, skin inflammation, and a chronic-recurrent course [3]. Its pathophysiology includes alterations in the skin barrier, immune dysfunction, and genetic susceptibility [4]. It is becoming increasingly evident that the implications of AD cannot be fully comprehended from a biological point of view.

In the last decade, a paradigm shift has taken hold in the understanding of AD, recognizing it as a disease with significant functional, emotional, and social repercussions [5]. In pediatrics, these repercussions take on special significance as they coincide with critical stages of emotional, cognitive, and social development.

Persistent pruritus, visible lesions, and the need for ongoing care may interfere with children’s well-being, social interactions, and school participation [6]. Despite this recognition, a significant gap persists in clinical practice between the objective assessment of skin severity and the subjective experience of the patient and their family [7].

It is common for children with skin involvement considered mild to experience a significant deterioration in their quality of life, while others with more extensive disease demonstrate better adaptation. This discrepancy can lead to an underestimation of the disease’s actual impact and to incomplete therapeutic strategies, focused on controlling visible lesions but disconnected from psychosocial needs. In this context, it is essential to adopt conceptual frameworks that allow for understanding AD as a complex and multidimensional phenomenon.

The objective of this review is to analyze the psychosocial impact of AD in childhood, integrating available evidence on quality of life, sleep disturbances, emotional distress, social stigmatization, and family burden, based on contemporary conceptual models, as well as to discuss its implications for patient- and family-centered clinical practice in pediatric allergology.

By integrating the biopsychosocial and burden-of-disease frameworks with a practical clinical aid, this review aims to translate multidimensional evidence into a structured approach applicable to routine pediatric AD care, with particular relevance to pediatric allergy practice.

## 2. Materials and Methods

A narrative literature review was conducted using PubMed/MEDLINE, Scopus, and Web of Science. The literature search covered publications from January 2013 through May 2026, with the final search performed in May 2026. Searches combined keywords and MeSH terms including “atopic dermatitis,” “eczema,” “children,” “quality of life,” “psychosocial impact,” “sleep disturbance,” “mental health,” and “caregiver burden,” using combinations of these terms as appropriate.

Eligible publications were written in English or Spanish and included observational studies, clinical trials, systematic reviews, meta-analyses, and clinical guidelines addressing pediatric AD and at least one relevant psychosocial domain, including health-related quality of life, sleep disturbance, emotional or behavioral outcomes, social functioning, treatment-related burden, or caregiver and family burden. Pediatric populations from birth through 18 years of age were prioritized. Publications before January 2013 were excluded from the primary literature selection. Adult-only studies were not used as primary evidence for pediatric outcomes but were considered when they provided relevant conceptual or methodological context applicable to the biopsychosocial or burden-of-disease frameworks.

Study selection was conducted by the authors based on relevance to the predefined scope of the narrative review. Titles and abstracts were initially assessed for relevance, followed by full-text evaluation when necessary. Because this review was designed as a narrative rather than a systematic review, a prospective PRISMA-based screening log was not maintained; therefore, numerical counts of records identified, duplicates removed, records screened, and records excluded are not available. The selected literature was synthesized narratively according to the following domains: health-related quality of life, sleep disturbance, emotional and behavioral impact, social stigmatization, caregiver and family burden, and clinical implications. Evidence from these domains was subsequently interpreted within complementary biopsychosocial and burden-of-disease frameworks.

## 3. Psychosocial Impact of Pediatric AD

### 3.1. Models for Understanding the Complexity of Pediatric AD

Pediatric AD exemplifies the complexity of chronic disease, as its clinical manifestations alone do not fully capture its impact on the daily lives of affected children and their families [8]. In this context, conceptual frameworks that integrate disease pathophysiology with psychological, behavioral, and social dimensions may provide a more comprehensive understanding of disease burden.

The biopsychosocial model provides a useful framework for understanding pediatric AD by integrating biological, psychological, behavioral, and social dimensions of disease and care [9]. Biological manifestations such as pruritus, inflammation, and skin barrier dysfunction interact with psychological factors including stress, anxiety, coping, and emotional regulation, as well as with social influences such as family relationships, school participation, and stigma. The biological heterogeneity of AD further indicates that apparently similar clinical phenotypes may arise from different underlying pathophysiological mechanisms [10].

This framework helps explain the clinical heterogeneity observed in everyday practice, whereby children with similar levels of skin involvement may experience substantially different functional and emotional burdens. Importantly, effective control of skin inflammation may not necessarily result in a proportional improvement in quality of life. Persistent pruritus, sleep disturbance, or family stress may continue to contribute to disease burden even when cutaneous symptoms appear adequately controlled [9].

The burden-of-disease framework extends this perspective by considering the functional, emotional, social, and economic consequences of AD [5,8]. In pediatric populations, this burden encompasses not only cutaneous symptoms but also sleep disturbance, emotional effects on the child, daily treatment demands, disruption of family functioning, and school and work absenteeism. This framework highlights dimensions of disease burden that may be overlooked during conventional clinical assessment.

**Figure 1:**
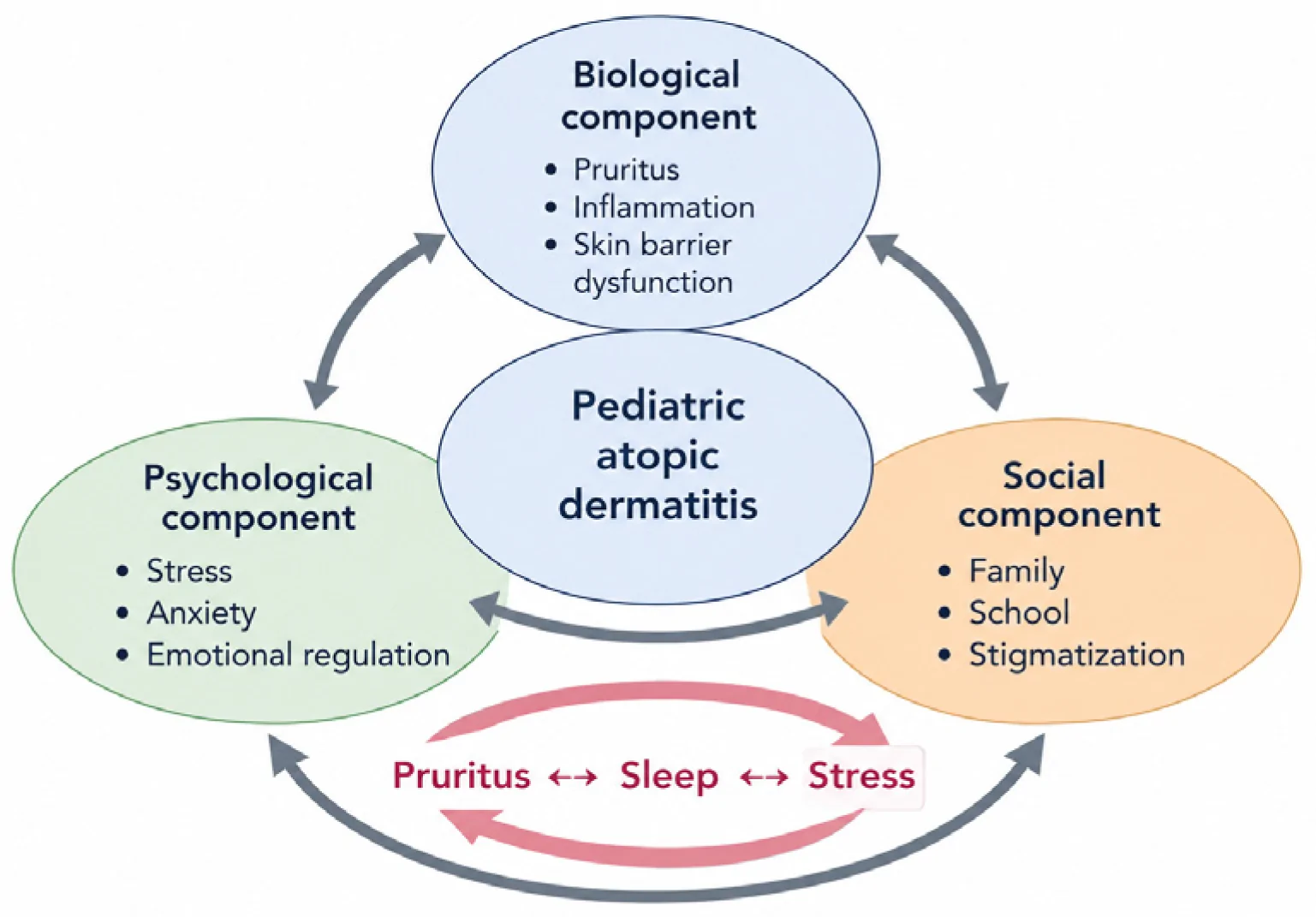
Triadic model of the impacts of pediatric AD. Pediatric AD stems from the complex interplay of biological, psychological and social components. Pruritus, inflammation, and skin barrier dysfunction interact with emotional stress and social determinants, generating a significant impact on the quality of life of both the child and their family. The pruritus–sleep–stress cycle acts as an amplifying mechanism of disease burden. The colors distinguish the biological, psychological, and social components, while the arrows indicate their bidirectional interactions.

Both models converge on the principle that skin-focused assessment alone is insufficient to capture the full burden of pediatric AD. Their integration provides a conceptual foundation for a more comprehensive and psychosocially informed understanding of the disease. Figure 1 illustrates the biopsychosocial model and the impact of AD in childhood.

The integrated representation presented here is intended as an educational synthesis of established biopsychosocial and burden-of-disease concepts rather than as a novel or independently validated theoretical model. The distinctive contribution of this review lies in translating these complementary frameworks into a clinically oriented approach to pediatric AD, with particular emphasis on pediatric allergy practice.

### 3.2. Health-Related Quality of Life: The Clinical–Subjective Discrepancy

Health-related quality of life (HRQoL) is one of the most affected domains in children with AD and constitutes an important component of the psychosocial impact of the disease [5].

Various studies using validated instruments, such as the Children’s Dermatology Life Quality Index (CDLQI) and the Infants’ Dermatitis Quality of Life Index (IDQoL), have demonstrated significant impairment across multiple domains, including physical, emotional, and social well-being, as well as participation in daily activities [11,12].

A clinically relevant finding is the imperfect correspondence between physician-assessed skin severity and the patient- or family-perceived impact of the disease [13]. Although greater clinical severity is generally associated with poorer quality of life, objective severity measures do not fully capture the individual burden experienced by all patients. This discrepancy highlights the complementary value of patient- and caregiver-reported outcomes in pediatric AD assessment. Prospective pediatric evidence further indicates that disease severity and changes in disease activity are associated with quality-of-life outcomes over time [14]. These findings highlight the limitations of traditional indices focused exclusively on the skin in capturing the full burden of the disease.

The subjective perception of itch intensity, duration, unpredictability, visibility, anatomical location, and nocturnal occurrence appears to influence quality of life more strongly than objective measures of disease severity. In addition, age, family dynamics, coping mechanisms, and psychosocial resources modulate the individual experience of disease burden.

These findings support the incorporation of patient- and caregiver-reported outcomes into pediatric AD assessment. The CDLQI and IDQoL assess the impact of AD on quality of life, whereas PO-SCORAD provides a patient- or caregiver-oriented assessment of disease severity [13]. Together, these tools complement physician-assessed measures by capturing dimensions of disease burden that may not be fully reflected by objective clinical examination. Several quality-of-life instruments have been developed and evaluated for infants, children, and adolescents with eczema, although their measurement properties and degree of validation vary [15]. A Spanish transcultural adaptation of the IDQoL has also been developed for children younger than four years with AD [16].

The multidimensional psychosocial impact of pediatric AD is summarized in Table 1.

### 3.3. Sleep Disturbances: A Pathophysiological and Psychosocial Axis

Sleep disturbance is common in children with AD and represents an important component of its psychosocial burden [17]. Pruritus, skin inflammation, and xerosis may interfere with sleep initiation and maintenance, leading to frequent awakenings, sleep fragmentation, and reduced restorative sleep [18].

**Figure 2:**
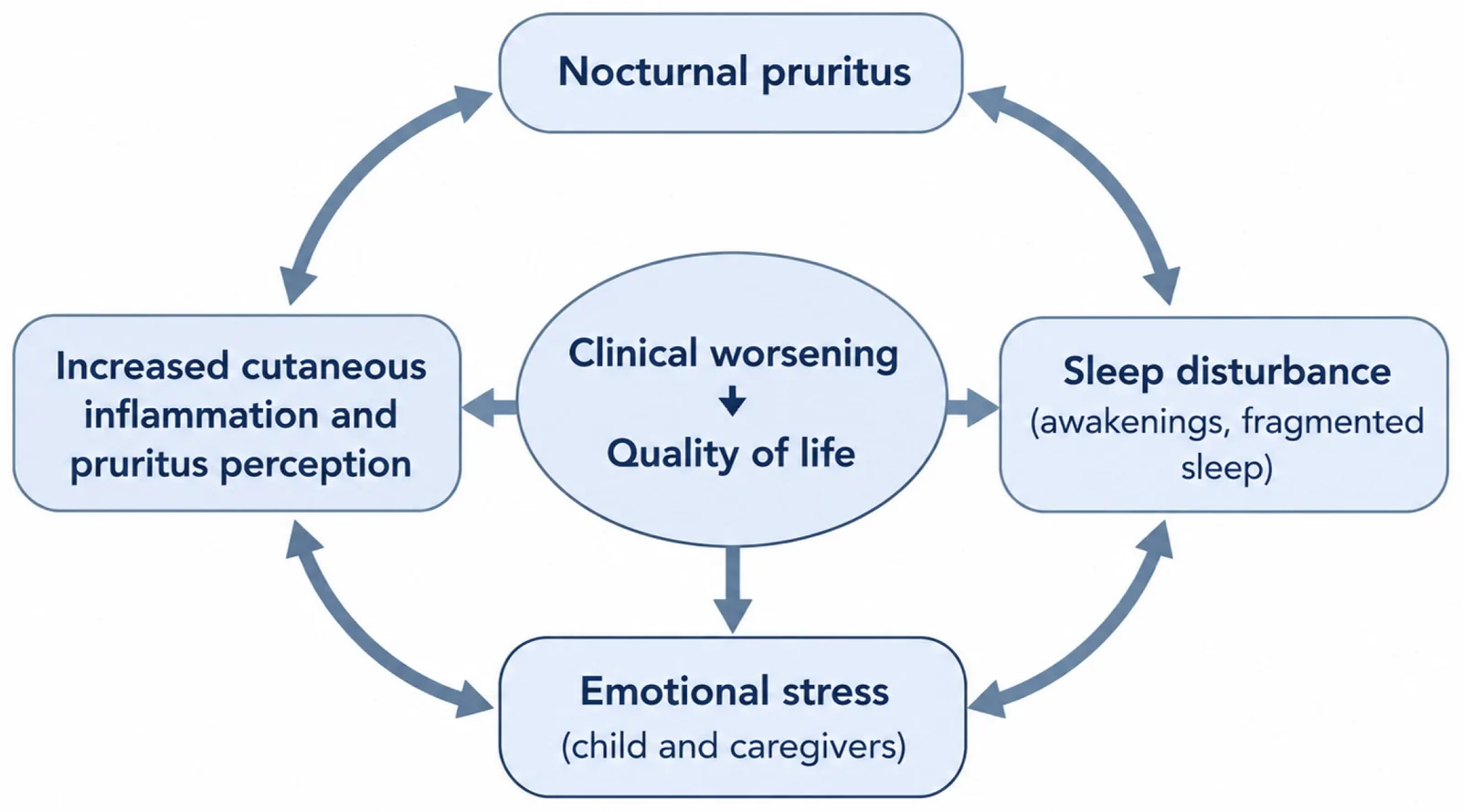
Bidirectional and cyclical interaction between pruritus, sleep, and stress in pediatric AD. Nocturnal pruritus contributes to sleep fragmentation and emotional distress in both the child and caregivers. Psychological stress may in turn influence the perception of pruritus and modulate neuroendocrine and immune pathways involved in AD, thereby contributing to overall disease burden. The arrows indicate the bidirectional and cyclical interactions among pruritus, sleep disturbance, emotional stress, and clinical worsening.

The relationship between sleep disturbance and AD appears to be bidirectional [19]. Nocturnal pruritus and worsening skin symptoms can disrupt sleep, while insufficient or fragmented sleep may contribute to psychological stress and altered neuroimmune regulation, potentially influencing pruritus perception and disease burden. These interactions may create a self-perpetuating cycle involving pruritus, sleep disturbance, and stress, as illustrated in Figure 2.

The consequences of sleep disturbance extend beyond nocturnal discomfort and may include daytime sleepiness, irritability, attention difficulties, and impaired daytime functioning [20,21]. Sleep disruption may also affect parents and caregivers, whose sleep can be interrupted by the child’s nocturnal symptoms and caregiving needs, thereby contributing to broader family burden [22].

### 3.4. Emotional and Behavioral Impact: The Skin as an Emotional Organ

The emotional and behavioral dimensions of AD are particularly relevant during childhood and adolescence, when self-image, emotional regulation, and social relationships are still developing. Children and adolescents with AD have an increased risk of psychological comorbidity compared with unaffected peers, including symptoms of depression and anxiety [23]. Longitudinal evidence further suggests an association between AD and internalizing mental health symptoms across childhood [24].

Persistent pruritus, recurrent flares, visible skin lesions, and the perceived lack of control over the disease may contribute to emotional distress and affect self-esteem and coping. These experiences should be interpreted within the broader biopsychosocial context of the child rather than as direct consequences of skin severity alone.

Psychological stress may influence AD through neuroendocrine and immune regulatory pathways, including mechanisms involving the hypothalamic–pituitary–adrenal (HPA) axis and stress-related inflammatory responses [25,26]. Qualitative evidence also identifies psychological stress as an important component of the lived experience of AD [27]. Together, these findings support a bidirectional relationship in which skin symptoms may contribute to emotional distress, while psychological stress may influence the perception and experience of disease.

Early recognition of clinically relevant emotional or behavioral concerns may facilitate further assessment and timely supportive interventions as part of comprehensive pediatric AD care.

### 3.5. Psychosocial Development and Social Stigmatization

Visible skin lesions may contribute to stigmatization and negative self-perception in children with AD. Social responses to visible disease may include staring, unwanted comments, avoidance, teasing, or bullying, with potential consequences for self-esteem and social interactions [28].

The psychosocial impact of visible skin disease may vary according to age and developmental stage. As children become increasingly aware of body image, peer relationships, and social acceptance, concerns related to appearance and perceived stigma may contribute to social withdrawal, avoidance of group activities, and difficulties in psychosocial adaptation [28,29].

These experiences may extend beyond the child and influence family concerns regarding social acceptance and participation in school and other age-appropriate activities. From a clinical perspective, recognizing stigma and social difficulties as part of the broader disease burden may help identify children and families who require additional support. Educational efforts directed toward children, caregivers, schools, and other relevant social environments may also help address misconceptions about AD and promote social participation.

### 3.6. Parental Anxiety and Family Burden: AD as a Family System Disease

Pediatric AD affects not only the child but also the broader family system. Its chronic, relapsing–remitting, and unpredictable course may place substantial demands on caregivers in terms of time, emotional resources, sleep, and daily disease management.

Caregivers frequently assume responsibility for treatment application, symptom monitoring, trigger identification, and ongoing treatment-related decisions. Qualitative evidence has identified the practical complexity of childhood eczema management and multiple caregiver-perceived barriers and facilitators to treatment adherence [30].

The burden of pediatric AD may also affect caregiver emotional well-being and family functioning. Caregivers report stress, sleep disruption, emotional exhaustion, and feelings of being overwhelmed by the demands of disease management [31]. International evidence further demonstrates that the impact of pediatric AD extends across multiple aspects of family life and is greater in families of children with more severe disease [32].

Topical corticosteroid phobia represents an additional treatment-related concern. Negative beliefs and concerns regarding topical corticosteroid use are common among patients with AD and their caregivers and may be associated with poorer treatment adherence. However, reported prevalence varies considerably according to the definitions and assessment methods used across studies [33].

Caregiver-related factors may also influence day-to-day disease management. Parental self-efficacy—the caregiver’s confidence in managing childhood eczema—represents a measurable component of family-centered care and may be assessed using validated instruments developed specifically for this purpose [34]. Recognizing caregiver burden, treatment concerns, and confidence in disease management may therefore provide clinically relevant information beyond assessment of the child’s skin disease alone.

### 3.7. Clinical Implications: Toward a Patient- and Family-Centered Approach

Clinical management of pediatric AD has traditionally emphasized the severity of cutaneous manifestations. However, contemporary care increasingly recognizes that disease burden extends beyond visible skin findings and includes health-related quality of life, sleep disturbance, treatment burden, adherence, emotional well-being, and family impact. These dimensions should complement, rather than replace, objective assessment of disease activity and severity.

Validated instruments may facilitate the assessment of dimensions of disease burden that are not fully captured during routine skin examination. The Children’s Dermatology Life Quality Index (CDLQI) and Infants’ Dermatitis Quality of Life Index (IDQoL) assess the impact of AD on quality of life, whereas PO-SCORAD provides a patient- or caregiver-oriented assessment of disease severity [13]. Family-oriented instruments, such as the Dermatitis Family Impact (DFI), can additionally help characterize caregiver and household burden [35]. These tools may support more structured communication between clinicians, patients, and caregivers and help identify areas requiring further assessment. Selected instruments relevant to the assessment of patient- and family-reported burden in pediatric AD are summarized in Table 2.

This broader assessment is consistent with current guidance for childhood AD. The National Institute for Health and Care Excellence (NICE) recommends that the psychological well-being and quality of life of children with atopic eczema and their families be discussed and recorded during eczema consultations, including the effects of disease on everyday activities and sleep. NICE also emphasizes that physical disease severity does not necessarily correspond directly to its impact on quality of life [36].

Contemporary European guidelines also support an age-appropriate, stepped-care approach to pediatric AD. The 2025 EuroGuiDerm living guideline provides specific treatment recommendations and a stepped-care plan for children and adolescents, including guidance regarding escalation to systemic therapy when appropriate [37].

Psychosocial burden should not be interpreted as an isolated indication for systemic therapy. Rather, persistent impairment in quality of life, sleep, daily functioning, or treatment adherence may provide complementary clinical information that prompts reassessment of disease control, adherence, treatment burden, and unmet therapeutic needs. From an allergy perspective, the EAACI biologicals guideline for dupilumab in moderate-to-severe AD further supports a structured, age-specific approach when advanced therapy is being considered [38]. Patient- and family-reported outcomes should therefore be interpreted alongside objective disease severity, previous treatment response, adherence, and other relevant clinical factors.

Supportive interventions may also form part of comprehensive care when clinically relevant psychosocial concerns are identified. Therapeutic patient and caregiver education may improve disease understanding, self-management skills, and treatment implementation. A systematic review and meta-analysis of randomized controlled trials of pediatric AD education programs found improvement in disease severity, whereas pooled effects on quality-of-life measures were not statistically significant; substantial heterogeneity was observed across several outcomes [39]. A separate meta-analysis of health education interventions in children with AD also reported improvements in disease severity and selected quality-of-life outcomes [40].

Psychological and behavioral support may also be considered in selected patients, particularly when emotional distress, maladaptive coping, sleep disturbance, or treatment-related difficulties persist despite appropriate medical management. A Cochrane review of psychological and educational interventions in children with AD found some evidence that educational interventions may improve disease severity and quality of life, while emphasizing the limited number, heterogeneity, and methodological limitations of the available studies [41].

Accordingly, multidisciplinary assessment or psychological support may be considered when clinically significant or persistent psychosocial concerns are identified, according to the individual needs of the child and family. Such interventions should complement, rather than replace, evidence-based dermatologic and allergy management.

To facilitate the clinical application of the domains identified in this review, the authors developed a proposed clinical aid based on recurring psychosocial dimensions identified across the reviewed literature and the conceptual frameworks discussed above. This tool has not undergone formal validation and is intended to support, rather than replace, individualized clinical assessment. The proposed domains, key clinical questions, and suggested tools or actions are summarized in Table 3.

## 4. Discussion

The psychosocial burden of pediatric AD is increasingly recognized; however, important gaps in the evidence remain. Much of the available literature is cross-sectional, limiting the ability to establish causal or temporal relationships. Although associations between AD and sleep disturbance, emotional and behavioral difficulties, and caregiver burden are well documented, the directionality and mechanisms underlying these relationships remain incompletely understood.

Considerable heterogeneity across studies further limits the interpretation and comparability of findings. Differences in study populations, definitions of disease severity, psychosocial outcomes, and measurement instruments make it difficult to translate the available evidence consistently into clinical practice. Moreover, although the overall psychosocial burden of AD is well established, evidence regarding the effectiveness of targeted interventions—such as sleep-focused strategies, therapeutic education, and psychological support—remains limited.

Developmental and contextual factors also require further investigation. The psychosocial experience of AD may differ substantially according to age, developmental stage, family dynamics, socioeconomic context, and social environment. Clinical–subjective discordance, in which patient- or family-perceived burden is disproportionate to physician-assessed disease severity, is particularly relevant, yet the factors underlying this discrepancy remain insufficiently characterized.

The biopsychosocial and burden-of-disease models provide complementary frameworks for understanding these complex interactions. Their integration may help clinicians move beyond skin-focused assessment toward a more comprehensive evaluation of the child and family. Future research should prioritize longitudinal studies, structured psychosocial interventions, and integrated care models to determine how these approaches influence quality of life, family burden, and clinical outcomes.

This review also has limitations. Its narrative design does not provide the methodological rigor or reproducibility of a systematic review and may be subject to selection bias in the identification and interpretation of relevant literature. In addition, although priority was given to pediatric evidence, selected adult studies were considered when they provided relevant conceptual or methodological context; therefore, not all findings can be assumed to be directly generalizable to children.

Finally, psychosocial burden should not be regarded solely as a consequence of AD, but as a potential modifier of the disease experience and response to treatment. Recognizing the bidirectional relationships among skin disease, sleep, emotional well-being, social functioning, and caregiver burden supports a more integrated, patient- and family-centered approach to pediatric AD.

## 5. Conclusions

Pediatric AD extends well beyond the skin, with substantial and interrelated effects on emotional well-being, sleep, social functioning, and family life. The bidirectional interactions among biological, psychological, and social factors support the need for a multidimensional approach to assessment and management. Particular attention should be given to clinical–subjective discordance, sleep disturbance, and caregiver burden, which may be underestimated during routine clinical evaluation. Integrating validated patient- and family-reported outcomes into clinical practice may facilitate more comprehensive and individualized care. Future research should prioritize longitudinal studies and structured interventions designed to determine whether addressing psychosocial burden improves both quality of life and clinical outcomes in children with AD.

## Figures and Tables

**Table 1:** Main dimensions and potential consequences of the psychosocial burden of pediatric atopic dermatitis.

Dimension	Manifestations	Potential Consequences
Quality of life	Activity limitation, persistent discomfort	Functional impairment and reduced well-being
Sleep	Nocturnal pruritus, frequent awakenings, fragmented sleep	Behavioral problems
Emotional	Anxiety, irritability	Psychosocial impairment
Social	Stigma, teasing, social withdrawal	School difficulties
Family	Caregiver stress, sleep loss, treatment burden, corticophobia	Poor adherence

**Table 2:** Selected instruments for assessing patient- and family-reported burden in pediatric atopic dermatitis.

Domain	Instrument	Intended Age/Respondent
Quality of life	CDLQI	Children aged 4 to <16 years; child-reported
Quality of life	IDQoL	Infants and young children; caregiver-reported
Subjective severity	PO-SCORAD	Children/caregivers; patient- or caregiver-reported
Sleep	Parental questionnaires	Age-dependent; usually caregiver-reported
Family burden	Dermatitis Family Impact (DFI)	Caregivers

Abbreviations: CDLQI, Children’s Dermatology Life Quality Index; IDQoL, Infants’ Dermatitis Quality of Life Index; PO-SCORAD, Patient-Oriented SCORAD; DFI, Dermatitis Family Impact.

**Table 3:** Proposed clinical aid for multidimensional assessment in pediatric AD.

Domain	Key Clinical Questions	Suggested Tools/Actions
Symptom burden beyond skin severity	Is pruritus persistent, nocturnal, or unpredictable? Does the perceived burden appear disproportionate to visible skin severity?	Clinical inquiry; PO-SCORAD
Sleep	Are there difficulties initiating sleep, frequent awakenings, nocturnal scratching, or daytime fatigue?	Sleep history; caregiver report
Emotional and behavioral well-being	Is there anxiety, irritability, low mood, emotional distress, or behavioral change?	Clinical inquiry; further assessment when concerns are identified
Social functioning	Are there school difficulties, stigma, bullying, social withdrawal, or limitations in age-appropriate activities?	Child and/or caregiver interview
Caregiver and family burden	Is there caregiver stress, treatment fatigue, sleep loss, or topical corticosteroid phobia?	DFI; caregiver discussion
Multidisciplinary needs	Are psychosocial concerns clinically significant, persistent, or interfering with disease management or daily functioning?	Consider psychological or multidisciplinary support

## Data Availability

No new data were created or analyzed in this study. Data sharing is not applicable to this article.

## Abbreviations

The following abbreviations are used in this manuscript:

AD	Atopic dermatitis
CDLQI	Children’s Dermatology Life Quality Index
DFI	Dermatitis Family Impact
HRQoL	Health-related quality of life
IDQoL	Infants` Dermatitis Quality of Life Index
PO-SCORAD	Patient-Oriented SCORAD
SCORAD	SCORing Atopic Dermatitis
HPA	Hypothalamic–pituitary–adrenal
